# Mining *Late Embryogenesis Abundant* (LEA) Family Genes in *Cleistogenes songorica*, a Xerophyte Perennial Desert Plant

**DOI:** 10.3390/ijms19113430

**Published:** 2018-11-01

**Authors:** Blaise Pascal Muvunyi, Qi Yan, Fan Wu, Xueyang Min, Zhuan Zhuan Yan, Gisele Kanzana, Yanrong Wang, Jiyu Zhang

**Affiliations:** State Key Laboratory of Grassland Agro-ecosystems; Key Laboratory of Grassland Livestock Industry Innovation, Ministry of Agriculture; College of Pastoral Agriculture Science and Technology, Lanzhou University; Lanzhou 730000, China; muvunyi14@lzu.edu.cn (B.P.M.); yanq16@lzu.edu.cn (Q.Y.); wuf15@lzu.edu.cn (F.W.); minxy15@lzu.edu.cn (X.M.); yanzhzh16@lzu.edu.cn (Z.Z.Y.); giskanzana@gmail.com (G.K.)

**Keywords:** *Cleistogenes songorica*, LEA proteins, gene expression analysis, abiotic stresses

## Abstract

Plant growth and development depends on its ability to maintain optimal cellular homeostasis during abiotic and biotic stresses. *Cleistogenes songorica*, a xerophyte desert plant, is known to have novel drought stress adaptation strategies and contains rich pools of stress tolerance genes. Proteins encoded by *Late Embryogenesis Abundant* (LEA) family genes promote cellular activities by functioning as disordered molecules, or by limiting collisions between enzymes during stresses. To date, functions of the *LEA* family genes have been heavily investigated in many plant species except perennial monocotyledonous species. In this study, 44 putative *LEA* genes were identified in the *C. songorica* genome and were grouped into eight subfamilies, based on their conserved protein domains and domain organizations. Phylogenetic analyses indicated that *C. songorica* Dehydrin and LEA_2 subfamily proteins shared high sequence homology with stress responsive Dehydrin proteins from Arabidopsis. Additionally, promoter regions of *CsLEA_2* or *CsDehydrin* subfamily genes were rich in G-box, drought responsive (MBS), and/or Abscisic acid responsive (ABRE) *cis*-regulatory elements. In addition, gene expression analyses indicated that genes from these two subfamilies were highly responsive to heat stress and ABA treatment, in both leaves and roots. In summary, the results from this study provided a comprehensive view of *C. songorica* *LEA* genes and the potential applications of these genes for the improvement of crop tolerance to abiotic stresses.

## 1. Introduction

Abiotic stresses from increasing temperature or salinity can disrupt optimal plant performance and cause significant crop yield losses [1]. To maintain proper homeostasis for normal growth, plants have evolved multiple ways to combat harsh environments by mobilizing a wide spectrum of stress responsive genes [2]. For example, proteins encoded by the *late embryogenesis abundant* (LEA) family genes are known to play defensive roles in plants during abiotic stresses [3,4]. The *LEA* family genes were first studied in cotton seed at the late phases of seed development [4]. The *LEA* family genes were later identified in various tissues of many other plant species and the proteins encoded by these genes were shown to be important during cold, drought and/or high salinity stresses [5,6]. LEA proteins are not plant specific, they are also found in invertebrates, fungi and bacteria [7,8]. Typical LEA proteins are highly hydrophilic due to high contents of charged amino acid residues, as well as amino acids like threonine, serine and alanine residues in their sequences [9]. It was suggested that LEA proteins have molecular shield functions [10] and are capable of abating protein aggregation and preventing enzyme degradation [11], thereby promoting proper cellular homeostasis during stresses [8,12]. LEA proteins are also flexible proteins which can undergo conformational changes and interact with other macromolecules including proteins, membranes and/or nucleic acids during different adverse stress conditions [10].

LEA proteins are divided into at least eight distinct subfamilies, based on their conserved protein domains in the Pfam database: LEA (1–6), Dehydrin and Seed Maturation Protein (SMP) [13]. Motif structures within subfamily genes are mostly conserved, except the genes in the *LEA_2/LEA5C* subfamily [9]. In addition, proteins in the LEA_2/LEA5C subfamily are known to have other non-canonical LEA protein properties like high hydrophobicity and at least one atypical LEA domain known as Water stress and Hypersensitive response (WHy) domain. The presence of atypical LEA domain(s) in LEA proteins indicate that these proteins may function differently from typical LEA proteins [14,15,16]. Proteins in the Dehydrin subfamily are featured with at least one K-segment, a 15 amino acid residue rich in lysine (i.e., EKKGIMDKIKEKLPG) and can function like chaperones to protect peripheral membrane and proteins during dehydration [2,17,18,19,20]. Numerous earlier studies have demonstrated that the *LEA* family genes are potential abiotic stress responsive genes, important for enhancing plant stress tolerance. For instance, transgenic Arabidopsis [21,22], maize [23], alfalfa [24] bacteria [25], yeast and tobacco [26] expressing different *LEA* genes exhibited improved abiotic stress tolerance compared with their respective wild-type plant, bacteria or yeast.

*C. songorica* is a xerophyte C4 desert plant distributed widely in the wild lands in the northwest part of China, with an annual precipitation of about 100 mm [27]. Previous genome-wide surveys of *LEA* family genes were done for multiple plant species except perennial monocotyledonous species. In this study, we investigated the *LEA* family genes in *C. songorica* and analyzed the responses of four selected *LEA* genes to heat stress or abscisic acid (ABA) treatment in leaves and shoots. Results from this study provide new information on the evolution of the LEA family proteins, protein structures and potential applications of these genes for the improvement of crop tolerance against abiotic stresses.

## 2. Results

### 2.1. Identification of CsLEA Genes and Phylogenetic Analysis

**Figure 1:**
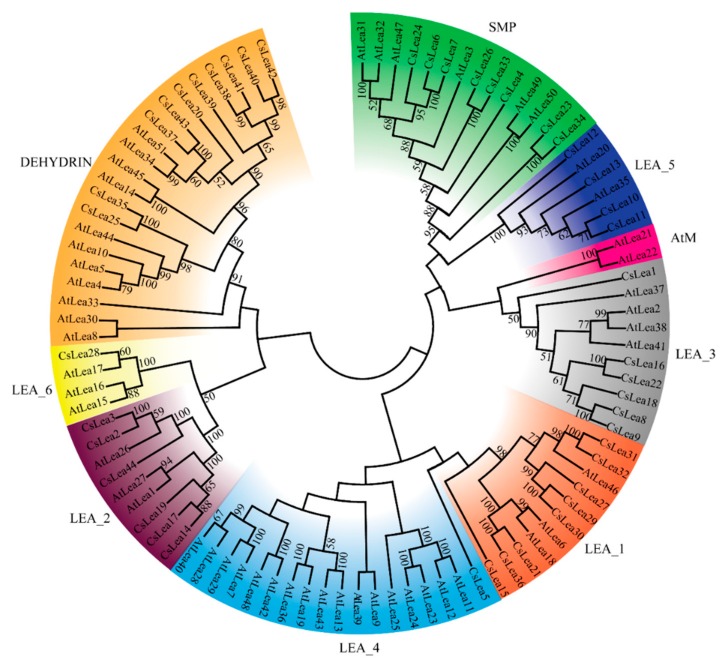
Phylogenetic analysis of *C. songorica* LEA proteins. Full-length amino acid sequences of the 44 CsLEA proteins were analyzed using the unrooted method in the ClustalW software.

A total of 44 putative *C. songorica* LEA proteins were identified in this study (Table S1). These proteins were named from CsLEA1 to CsLEA44 and grouped into eight different subfamilies (Figure 1): CsLEA_1, CsLEA_2/LEA5C (Battaglia classification), CsLEA_3, CsLEA_4, CsLEA_5, CsLEA_6, SMP and Dehydrin, based on their Pfam conserved protein domains and their homology with the published LEA proteins of *A. thaliana* [28]. Two atypical LEA stress related domains, Water Stress and Hypersensitive response (WHy) and LEA14-like desiccation related protein (COG5608), were detected in the proteins from CsLEA_2 subfamily (Figure S1). 

### 2.2. Structures, Physiochemical Properties and Subcellular Localizations of CsLEA Proteins

Most proteins within the same family exhibited similar structures and properties (Table S1). Over one third of the CsLEA proteins were classified as unstable proteins with an instability index value higher than 40. Furthermore, this property varied significantly among the proteins within the Dehydrin subfamily, ranging from 3.83 to 56.11. All the proteins in the LEA_5 subfamily showed instability index values greater than 47 and were considered as the most unstable and/or potentially disordered proteins [29,30,31]. The GRAVY (grand average of hydropathicity index) values of more than 90% CsLEA proteins were below 0, stressing that CsLEA proteins are likely to have low hydrophobicity features. CsLEA_2 subfamily proteins were the most hydrophobic proteins, while Cs_LEA5 proteins were the highest hydrophilic proteins. These results are consistent with previous studies [28,32] and reinforce the structural disordered properties of LEA proteins by which they are capable of interacting with other molecules and mitigating the collision of enzymes during plant stress conditions [11] 

### 2.3. Gene and Motif Structure Analyses

**Figure 2:**
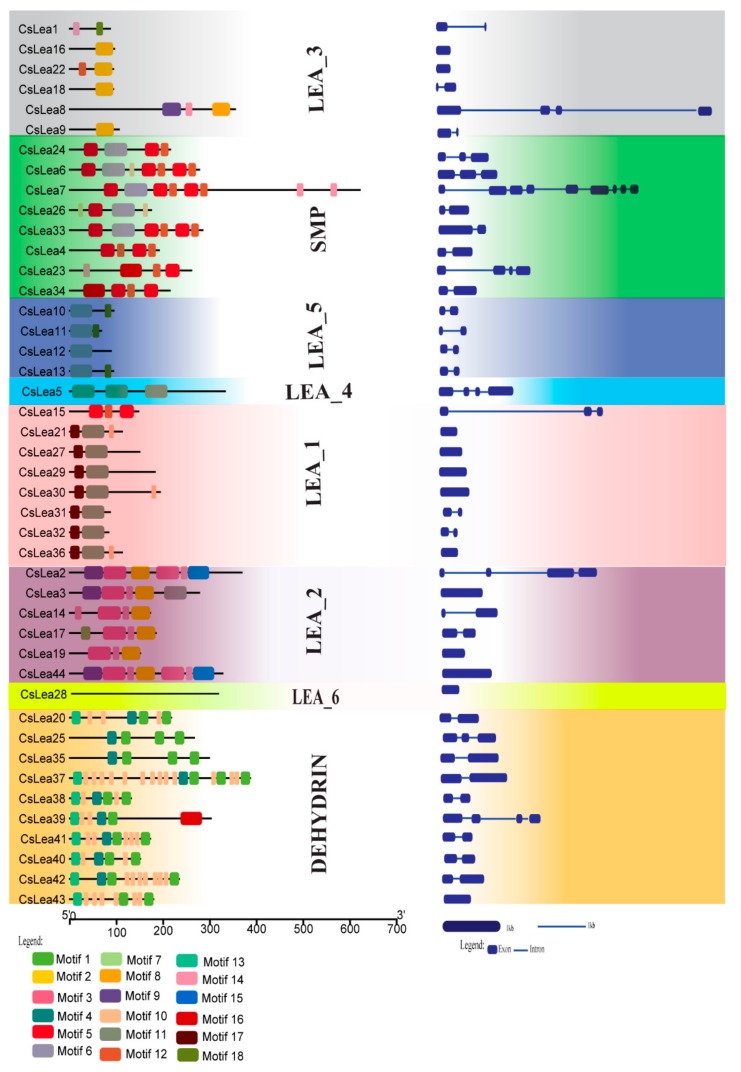
Motif structure and exon–intron organizations of the 44 CsLEA genes. The 18 motifs discovered in this study are shown on these *CsLEA* genes (**left**). The blue boxes represent exons and the blue lines represent introns (**right**).

*LEA* genes within the same subfamily showed similar exon and intron architectures (Figure 2, right panel). Further investigation of structures of paired genes at the short-end branches in the phylogenetic tree revealed that six of them (e.g., CsLEA 43–37, 35–25, 2–3, 8–9, 23–34 and 6–7) might have experienced exon-intron gain/loss events during their evolutionary history. Similar situations have been reported for Brassica *LEA* genes [21,33]. In total, 18 motifs were identified in 43 CsLEA proteins (Figure 2, left panel). No motif was found in CsLEA28 protein. Except for LEA_2 and LEA_3 subfamily proteins, motif structures and compositions were nearly identical among the proteins in the same subfamily, but differed significantly between the proteins belonging to different subfamilies, implying functional specificities of different CsLEA subfamily proteins [14,34].

### 2.4. Sequence Alignment of *C. Songorica* and Arabidopsis Dehydrin Proteins

**Figure 3:**
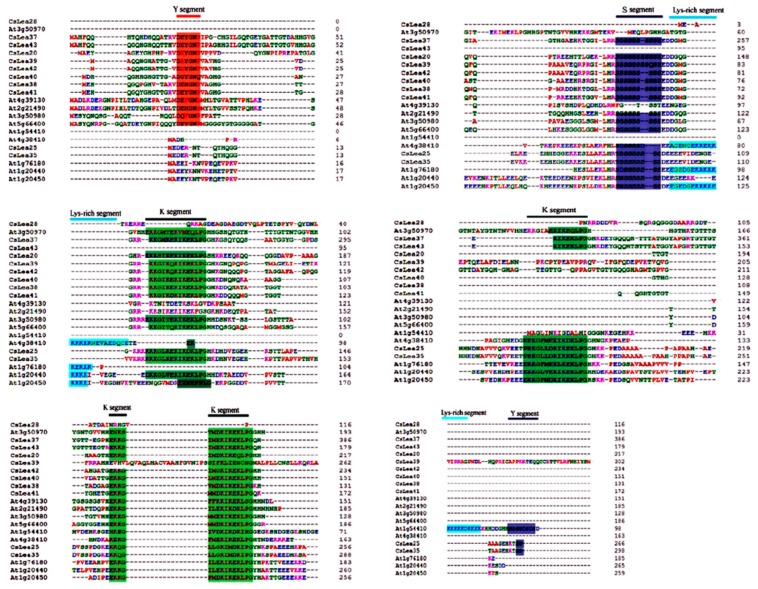
Multiple sequence alignment using *C. songorica* and Arabidopsis Dehydrin protein sequences. The identified Y segment, K segment and S segment are indicated by different colors. Y segment = red, K segment = green and S segment = purple.

Multiple sequence alignment using *C. songorica* Dehydrin protein sequences and their Arabidopsis counterparts revealed the conservation stress response related segments including Y, K and S segments (YKS) in *C. songorica* Dehydrin proteins (Figure 3).

### 2.5. Cis-Regulatory Element in *C. Songorica* LEA Gene Promoters

*Cis*-regulatory elements control expression patterns of stress responsive genes in various tissues and organs. These elements are located upstream of gene coding sequences and provide binding sites for transcription factors (TFs) [35]. More than three G-box *cis*-elements were recorded for each *Dehydrin*, *LEA_2* and *SMP* genes and nearly more than two MBS elements were detected for each *Dehydrin* and *LEA_2* subfamily gene (Table 1).

### 2.6. Chromosomal Mapping of CsLEA Genes

**Figure 4:**
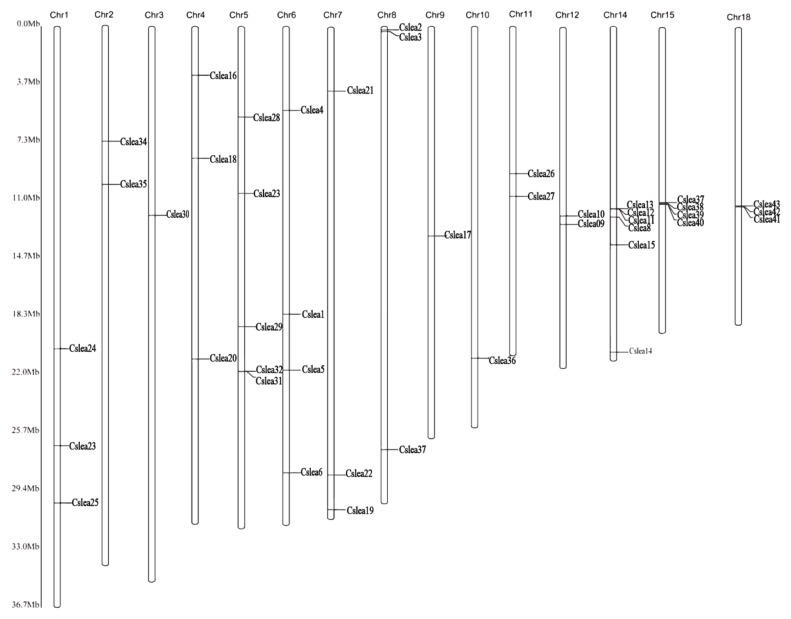
Locations of the 44 *CsLEA* genes on 15 chromosomes of *C. songorica*.

*C. songorica* genome has in total twenty chromosomes. Positions of the 44 *CsLEA* genes on 15 different *C. songorica* chromosomes were estimated (Figure 4). Genes from the same subfamily were mostly found on different chromosomes, suggesting a strategy to exert their functions across the whole *C. songorica* genome. However, genes in the *LEA_5* and *Dehydrin* subfamily were mostly found in clusters on the 14th, 15th and 18th chromosome.

### 2.7. Gene Expression Analysis qRT-PCR Validation

**Figure 5:**
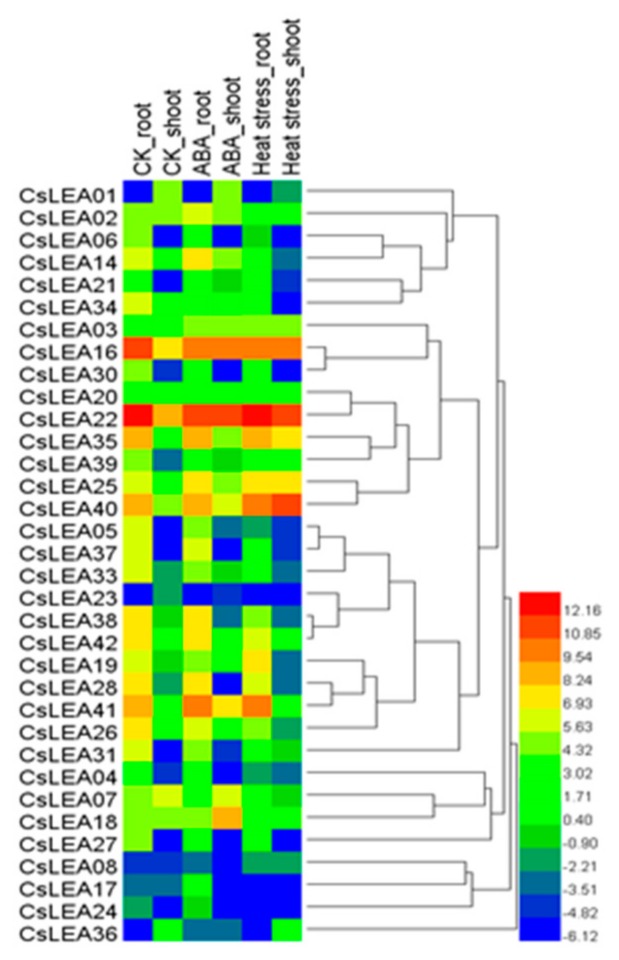
Hierarchical clustering of *CsLEA* gene expression profiles in root and shoot tissues after 24 h heat or ABA treatment. The log transformed values for the relative expressions of *CsLEA* genes were used for the hierarchical clustering analysis. The blue scale means low transcript expression and the red scale means high transcript expression.

The expression levels of the *CsLEA 14*, *CsLEA 19*, *CsLEA 37* and *CsLEA 38* genes were induced after 24 h of ABA or heat treatment but were not tissue specific (Figure 5). To validate results from expression profile analysis, qRT-PCR was carried out for *CsLEA 14*, *CsLEA 19* (from the *LEA_2* subfamily) and for *CsLEA37* and *CsLEA 38* (from the *Dehydrin* subfamily) as these genes showed a relatively high number of stress related *cis* acting elements and motifs.

**Figure 6:**
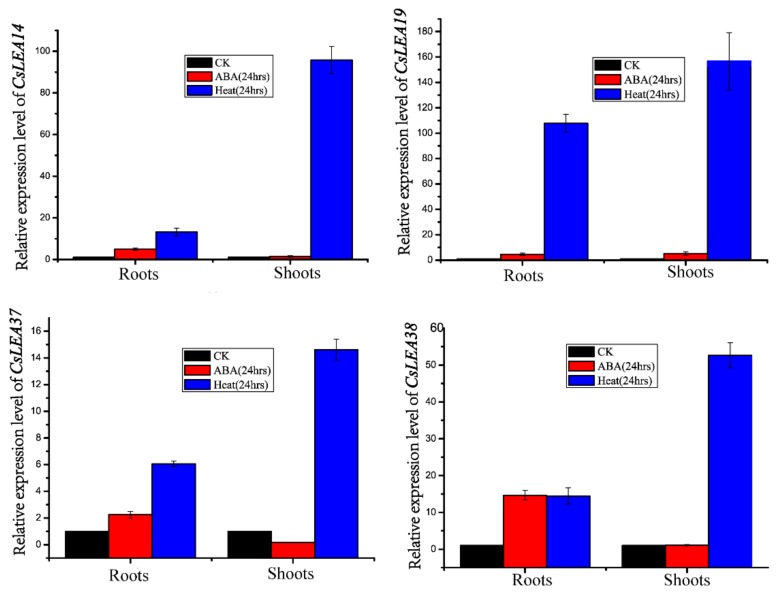
Hierarchical clustering of *CsLEA* gene expression profiles in root and shoot tissues after 24 h heat or ABA treatment. The log transformed values for the relative expressions of *CsLEA* genes were used for the hierarchical clustering analysis. The blue scale means low transcript expression and the red scale means high transcript expression.

We carried out qRT-PCR analyses using *CsLEA14* and *CsLEA19* (*LEA_2* subfamily), and *CsLEA37* and *CsLEA 38* (*Dehydrin* family). Results showed that after 24 h heat treatment the expression levels of these four genes up-regulated by 156.5 (*CsLEA 19*), 95.8 (*CsLEA14*), 52.6 (*CsLEA38*) and 14.6 fold (*CsLEA37*) in *C. songorica* leaves compared with the untreated plant leaf samples. The expression levels of these four genes were slightly up-regulated after the ABA treatment, especially *CsLEA 38* (14.6 fold, Figure 6).

## 3. Discussion

### 3.1. Phylogeny Analysis and Protein Sequence Analysis

Genome surveys of LEA subfamily proteins (e.g., LEA_1 to LEA_6, SMP and DEHYDRIN) were done for *A. thaliana* [28], rice [36] and maize [37]. *C. songorica* is a perennial monocotyledonous desert plant with a high tolerance for drought stress. Investigation of stress responsive proteins encoded by *LEA* family genes in a desert plant should benefit crop improvement for drought and other abiotic stresses. Phylogenetic analysis grouped the CsLEA proteins into eight different subfamilies. Several *C. songorica* and Arabidopsis Dehydrin subfamily proteins were clustered together with high bootstrap values, which implied potential significant functional similarities between *C. songorica* and Arabidopsis DEHYDRIN proteins. Many of the Arabidopsis Dehydrin proteins are known as stress regulatory proteins such as RAB18 (*AtLea51*) and COR47 (*AtLea4*), two ABA and cold inducible proteins [38,39], ERD14 (*AtLea4*) and ERD10 (*AtLea5*), two disordered chaperon proteins [40] and Dehydrin Xero2 (*AtLea33*), a disordered cold responsive protein with membrane binding activity [41]. 

Sequence alignment using CsDehydrin proteins and their Arabidopsis counterparts revealed that all *C. songorica* Dehydrin proteins contained YKS segments but lacked the lysine rich segment. The K segment is critical for the formation of structural disordered alpha-helical compounds that can enhance bindings between proteins and their targeted molecules [10,42]. The presence of the K segment in *C.songorica* DEHYDRIN proteins emphasizes their role in limiting aggregation of molecules and thence promoting proper cellular homeostasis during dehydration stresses [11]. Additionally, the detection of the S segment in the *C. songorica* DEHYDRIN protein also suggests their implication in enhancing plant tolerance against abiotic stresses through protein phosphorylation, as previous studies indicated that the S segment participates in calcium binding through protein phosphorylation [43]. The findings above support the fact that disordered LEA proteins are flexible proteins, capable of adjusting their conformation to maintain proper cellular homeostasis during detrimental stress conditions [43,44].

### 3.2. Protein Domain Analysis

Two additional non-LEA conserved protein domains that are associated with stress response were detected in the CsLEA_2 subfamily proteins (Supplementary Figure S1). At least one WHy (Water stress and Hypersensitive response) [15,16] and one COG5608 domain (LEA14-like desiccation related protein) was spotted within each single protein sequence from the CsLEA_2 subfamily. The COG5608 domain was previously detected in the Arabidopsis LEA14 protein, a well characterized abiotic stress marker protein, which suggests that the CsLEA_2 subfamily proteins may function similarly to Arabidopsis LEA14 protein (*Atlea1*). On the other hand, a thorough functional characterization of the WHy domain has only been elucidated in a few bacterial genes, *dwhy1* [15] and *drwh* [45]. Studies in vivo indicated that *dwhy1* confers cold and freeze damage resistance. Furthermore, in *E. coli*, the function of *drwh* is related to oxidative stress tolerance and salinity stresses. Silencing this gene triggered reduced activity of antioxidant enzymes such as lactate dehydrogenase (LDH) malate dehydrogenase (MDH) [15,45]. All the *CsLea_2* subfamily genes contained the WHy domain which could explain the functional importance of this domain during low water availability in a typical desert grass, *C. songorica*.

### 3.3. C. songorica Gene Promoter and Gene Expression Analysis

ABA and stress responsive *cis*-regulatory elements, such as ABRE, MBS/MYB and G-Box were found to be abundant in the promoters of *C. songorica* Dehydrin and the *LEA_2* subfamilies genes. These regulatory elements are known to provide binding sites for transcription factors like ABEF (a member of the bZIPTFs family), BHLH and ERF for the transcription of downstream stress responsive genes [46]. Expression analysis of *CsLEA37* and *CsLEA38* (*DEHYDRIN* genes), and *CsLEA14* and *CsLEA19* (*LEA_2* subfamily genes) with qRT-PCR indicated that the expression levels of these four genes in root and shoot tissues were significantly up-regulated after the drought or ABA treatment. 

The relevant role of DEHYDRIN or the LEA_2 subfamily proteins during plant stress tolerance has been reported in earlier studies using various transgenic plants. For example, transgenic tobacco plant overexpressing the *CaLEA6* gene showed an enhanced dehydration and salt tolerance [47]. Additionally, sweet potato plants overexpressing the *IbLEA14* gene exhibited an improved salinity and dehydration tolerance [48]. A Foxtail millet plant overexpressing the *SiLEA4* gene displayed salt and drought resilience [49]. For DEHYDRIN proteins, transgenic Arabidopsis plant expressing a *Dehydrin* gene from an olive showed an enhanced osmotic stress tolerance [50]. Similarly, a wheat *Dhn-5* gene increased salinity and dehydration stress tolerance in transgenic Arabidopsis plants [51]. *C. songorica DEHYDRIN* and *LEA_2* gene transcripts accumulation during water deficit and ABA treatment, reinforcestheir functional importance under detrimental stress conditions.

## 4. Materials and Methods

### 4.1. Mining LEA Genes in the C. Songorica Genome

*C. songorica LEA* genes were mined based on their protein sequence homology with the previously published *A. thaliana* [28], *Oryza sativa* (rice) [36] and *Zea mays* (maize) [37] LEA protein sequences. The published full length *A. thaliana*, rice and maize LEA protein sequences or coding sequences were retrieved from (https://phytozome.jgi.doe.gov/pz/portal.html) [28,32]. The obtained LEA protein sequences were used as queries to blast search the whole *C. songorica* genome sequence retrieved from the BMK cloud: http://www.biocloud.net/ using a local blast tool [52,53]. 

The resulting non-redundant sequences were further examined with the Hidden Markov Model available in the Pfam database (http://pfam.sanger.ac.uk/search) [13] and then submitted to the SMART database (http://smart.embl-heidelberg.de/) [54] and the NCBI Conserved Domain Search database (http://www.ncbi.nlm.nih.gov/Structure/cdd/wrpsb.cgi) [55], respectively, to confirm CsLEA Pfam domain families. The obtained LEA nucleotide and protein sequences were then submitted to the Genbank to obtain respective accession numbers (Table S1). 

### 4.2. Multiple Sequence Alignment and Phylogenetic Analysis of CsLEA Family Proteins.

The alignment of the *C. songorica* and Arabidopsis LEA protein sequences was performed using the ClustalW software [56] in the MEGA 6 program with a default parameter setting. After sequence alignment and pair-wise deletion of gaps as previously described [57], a phylogenetic tree was constructed using the Neighbor Joining (NJ) algorithm with bootstrap analysis of 1000 trials [57,58]. Multiple sequence alignment and sequence homology analysis of *C. songorica* and Arabidopsis Dehydrin proteins were performed using the ClustalW algorithm embedded in the DNAMAN version 6 program as instructed (Lynnon Corporation, Quebec, Canada).

### 4.3. In Silico Analyses of CsLEA Proteins.

Determination of GRAVY (grand average of hydropathicity index) values and pI (theoretical isoelectric point) were carried out by the ProtParam Tool (web.expasy.org/protparam/) [59]. Protein Prowler Subcellular Localization Predictor version 1.2 (http://bioinf.scmb.uq.edu.au/pprowler_webapp_1–2/) 53 and TargetP1.1 (http://www.cbs.dtu.dk/services/TargetP/) servers [60] were used to predict the subcellular locations of *C. songorica* LEA proteins. All of the prediction servers were run under the default settings. To determine the conserved motifs in different *C. songorica* and Arabidopsis LEA proteins, protein sequences were analyzed using MEME (The Multiple Expectation Maximization for Motif Elicitation) platform (http://alternate.meme-suite.org/) [61]. MEME parameters were then customized to detect a maximum of 40 motifs with a width covering 6 to 50 amino acid residues.

### 4.4. Analysis of *Cis*-Regulatory Elements and Motifs

Sequences of 2000 bp from promoters of the 44 identified *C. songorica LEA* genes were analyzed for potential *cis*-regulatory elements and motifs by querying them through the PlantCARE database (http://bioinformatics.psb.ugent.be/webtools/plantcare/html/) [62]. Stress- and ABA-related *cis*-regulatory elements, including MYB binding site (drought responsive) [63], G-box (light inducible) [64], ABRE (Abscisic acid responsive) [65] and CGTCA-motif (Methyl jasmonate responsive) [66] were recorded. 

### 4.5. Plant Material Preparation and Transcriptomic Data Analysis

*C. songorica* seeds were sown in vermiculite medium supplied with 1/4 diluted Hoagland’s nutrient solution, pH 5.8. Growth chamber conditions were set at 75–80% relative humidity, 30/28 °C (day/night), and 16/8 h (day/night) light at 200 mmol photons m^−2^ s^−1^. One-month old seedlings were treated with 40 °C or with 100 μM ABA. Root and shoot tissue samples were collected at 0 and 24 h post the treatment and kept at −80 °C till RNA extraction. Three root and shoot samples were collected from each treatment. Total RNA was isolated from the samples using the Shengong RNA isolation kit as instructed (Shengong Ltd., Shanghai, China). RNA pools were constructed following Illumina sequencing guidelines and then sequenced conferring to RNA-seq procedure. In total 24 million 250-bp raw reads were produced from the 12 samples. To eliminate adapter sequences from raw reads, the FASTX version 0.0.13 toolkit (http://hannonlab.cshl.edu/fastxtoolkit/) was used. Additionally, the FastQC server tool (http://www.bioinformatics.babraham.ac.uk/projects/fastqc/) was utilized to assess the quality of sequences. The resulting clean reads were aligned with the *C. songorica* genome by means of Tophat v.2.0.10 server (http://tophat.cbcb.umd.edu/) [67] and the produced alignment files were used as Cufflinks inputs to create transcriptome assemblies [68]. The *C. songorica* gene expression levels were estimated based on fragments per kilobase of exon model per million mapped reads (FPKM) for root and shoot tissues. Following this, a random sampling model built on read count for each individual gene was applied to determine differentially expressed [69]. Gene expression levels were normalized with Pearson coefficients to generate hierarchical clustering with average linkage using HemI toolkit [70].

### 4.6. Gene Expressions Analysis.

The isolated RNA was then used to synthesize first strand cDNAs using an oligo dT primer and the cDNA synthesis kit (Shengong Ltd, Shanghai, China). The resulting cDNA samples were individually diluted to 100 ng/μL prior to qPCR using gene specific primers (Table S2). For each PCR reaction, three biological replicates with three technical replicates were each used. qPCR reactions were 40 cycles of 95 °C for 5 s, 60 °C for 15 s, and 72 °C for 34 s using a SYBR Green Master (Shengong Ltd, Shanghai, China). The relative gene expression levels were determined using the comparative ΔΔ*Ct* method [71]. The expression level of the *C. songorica GADPH* gene was used as an internal control. Two-way analysis of variance and Duncan’s multiple range test (DMRT) were used for multiple mean comparisons. SPSS (IBM Corp. 2013, IBM SPSS Statistics for Windows, Version 21.0, Armonk, NY, USA) was used to determine the significant differences between means (*p* < 0.005).

## 5. Conclusions

At a glance, this study methodically investigated at a genome wide level LEA proteins from a monocot perennial desert plant, *Cleistogenes songorica*. A total of 44 genes discovered were classified into eight different subfamilies and were found to be patchily spread over the *C. songorica* chromosomes. Analysis of the physio-chemical properties, motif and gene structure, homology and phylogenetic relationships detected that they were mostly similar within the same groups, but greatly differed among different subfamilies. Our study particularly explored CsLEA_2 and CsDEHYDRIN subfamilies proteins and elucidated their striking links with the regulatory mechanisms of plant abiotic stress tolerance. This study delivers a comprehensive summary of the evolution of the *C. songorica LEA* genes and some groundbreaking insights to the functional roles of this family that can be a critical foundation for crop abiotic tolerance improvement.

## Figures and Tables

**Table 1:** Stress-related *cis*-regulatory elements in 44 *C. songorica LEA* gene promoters.

CsLEA Subfamilies	Gene Names	Functional *cis*-Element Names and Sequences			
		MBS. (CGGTC)	G-Box (GTGCAT/CACGAC)	ABRE (GACACGTACGT)	CGTCA Motif
		Functions			
		Drought Responsive (MYB Binding Site)	Light Responsive	Abscisic Acid Responsive	MeJA Responsive
LEA_1	*CsLEA29*	3	1	1	0
	*CsLEA30*	2	3	1	4
	*CsLEA31*	4	1	1	0
	*CsLEA32*	2	2	1	0
	*CsLEA36*	4	5	2	2
LEA_2	*CsLEA2*	2	1	0	3
	*CsLEA3*	0	3	2	2
	*CsLEA14*	2	6	1	0
	*CsLEA17*	3	9	3	0
	*CsLEA19*	5	0	0	1
	*CsLEA44*	0	0	0	2
LEA_3	*CsLEA1*	4	2	0	2
	*CsLEA16*	1	2	0	3
	*CsLEA22*	1	3	1	3
	*CsLEA18*	8	3	3	0
	*CsLEA8*	0	7	1	2
	*CsLEA9*	3	0	0	0
LEA_4	*CsLEA5*	1	5	4	1
LEA_5	*CsLEA10*	0	2	0	1
	*CsLEA11*	0	3	0	3
	*CsLEA12*	2	7	1	1
	*CsLEA13*	2	1	3	5
LEA_6	*CsLEA28*	0	9	5	3
SMP	*CsLEA24*	0	4	1	0
	*CsLEA6*	2	0	2	0
	*CsLEA7*	2	6	0	0
	*CsLEA26*	1	5	1	1
	*CsLEA33*	0	5	1	4
	*CsLEA4*	2	1	0	0
	*CsLEA23*	2	1	0	0
	*CsLEA34*	1	4	0	1
	*CsLEA42*	0	3	1	2
	*CsLEA43*	0	0	1	5
Dehydrin	*CsLEA20*	4	5	0	0
	*CsLEA25*	4	2	0	1
	*CsLEA35*	3	8	2	1
	*CsLEA37*	0	6	3	4
	*CsLEA38*	2	10	2	3
	*CsLEA39*	3	1	0	1
	*CsLEA41*	2	0	1	2
	*CsLEA40*	0	2	0	1
	*CsLEA42*	0	3	1	2
	*CsLEA43*	0	0	1	5

## Supplementary Materials

Supplementary materials can be found at http://www.mdpi.com/1422-0067/19/11/3430/s1.
